# Supplementary material for: Modeling glioblastoma heterogeneity as a dynamic network of cell states
Source: Mol Syst Biol. 2021 Sep 16;17(9):e10105. doi: 10.15252/msb.202010105 (PMC8444284; doi:10.15252/msb.202010105)
Supplement: Supplementary file 6 — Source Data for Figure 5 [file MSB-17-e10105-s004.zip › Figure5A_sourcedata/GSEA_3071/hallmarks_stateI.GseaPreranked.1623742531165/HALLMARK_APICAL_SURFACE.html]

Details for gene set HALLMARK\_APICAL\_SURFACE[GSEA]

|  || Dataset | state13071 |
| Phenotype | NoPhenotypeAvailable |
| Upregulated in class | na\_neg |
| GeneSet | HALLMARK\_APICAL\_SURFACE |
| Enrichment Score (ES) | -0.21502115 |
| Normalized Enrichment Score (NES) | -0.6908464 |
| Nominal p-value | 0.8336252 |
| FDR q-value | 0.87582624 |
| FWER p-Value | 1.0 |
Table: GSEA Results Summary

  

Fig 1: Enrichment plot: HALLMARK\_APICAL\_SURFACE      
 Profile of the Running ES Score & Positions of GeneSet Members on the Rank Ordered List

  

| PROBE | GENE SYMBOL | GENE\_TITLE | RANK IN GENE LIST | RANK METRIC SCORE | RUNNING ES | CORE ENRICHMENT || 1 | THY1 |  |  | 94 | 1.114 | 0.1217 | No |
| 2 | CROCC |  |  | 528 | 0.490 | 0.0633 | No |
| 3 | CRYBG1 |  |  | 542 | 0.484 | 0.1242 | No |
| 4 | CX3CL1 |  |  | 828 | 0.373 | 0.0923 | No |
| 5 | GSTM3 |  |  | 1182 | 0.292 | 0.0303 | No |
| 6 | AFAP1L2 |  |  | 1247 | 0.278 | 0.0491 | No |
| 7 | ATP8B1 |  |  | 1280 | 0.272 | 0.0761 | No |
| 8 | GATA3 |  |  | 1417 | -0.250 | 0.0706 | No |
| 9 | SHROOM2 |  |  | 2230 | -0.360 | -0.1136 | No |
| 10 | DCBLD2 |  |  | 2407 | -0.393 | -0.1115 | No |
| 11 | APP |  |  | 2770 | -0.495 | -0.1490 | Yes |
| 12 | FLOT2 |  |  | 2803 | -0.502 | -0.0912 | Yes |
| 13 | GAS1 |  |  | 3009 | -0.607 | -0.0688 | Yes |
| 14 | BRCA1 |  |  | 3185 | -0.765 | -0.0169 | Yes |
| 15 | SULF2 |  |  | 3233 | -0.823 | 0.0795 | Yes |
Table: GSEA details [plain text format]

  

Fig 2: HALLMARK\_APICAL\_SURFACE: Random ES distribution      
 Gene set null distribution of ES for **HALLMARK\_APICAL\_SURFACE**
